# Ectopic and visceral fat deposition in aging, obesity, and idiopathic pulmonary fibrosis: an interconnected role

**DOI:** 10.1186/s12944-023-01964-3

**Published:** 2023-11-24

**Authors:** Xiaoyun Cheng, Shuhan Jiang, Boyu Pan, Wei Xie, Jie Meng

**Affiliations:** 1 Department of Pulmonary and Critical Care Medicine, The Third Xiangya Hospital of Central South University, Tongzipo Road 138, Yuelu District, Changsha, 410000 China; 2 Hunan Key Laboratory of Organ Fibrosis, Tongzipo Road 138, Yuelu District, Changsha, 410000 China; 3 Departments of Orthopedics, The Third Hospital of Changsha, Laodong West Road 176, Tianxin District, Changsha, 410000 China; 4 Department of Cardiology, Xiangya Hospital of Central South University, Furong Middle Road 36, Kaifu District, Changsha, 410000 China

**Keywords:** Idiopathic pulmonary fibrosis, Aging, Ectopic fat deposition, Inflammation, Visceral adipose tissue

## Abstract

Idiopathic pulmonary fibrosis (IPF) is considered an age-related disease. Age-related changes, along with other factors such as obesity, hormonal imbalances, and various metabolic disorders, lead to ectopic fat deposition (EFD). This accumulation of fat outside of its normal storage sites is associated with detrimental effects such as lipotoxicity, oxidative stress, inflammation, and insulin resistance. This narrative review provides an overview of the connection between ectopic and visceral fat deposition in aging, obesity, and IPF. It also elucidates the mechanism by which ectopic fat deposition in the airways and lungs, pericardium, skeletal muscles, and pancreas contributes to lung injury and fibrosis in patients with IPF, directly or indirectly. Moreover, the review discusses the impact of EFD on the severity of the disease, quality of life, presence of comorbidities, and overall prognosis in IPF patients. The review provides detailed information on recent research regarding representative lipid-lowering drugs, hypoglycemic drugs, and lipid-targeting drugs in animal experiments and clinical studies. This may offer new therapeutic directions for patients with IPF.

**Supplementary Information:**

The online version contains supplementary material available at 10.1186/s12944-023-01964-3.

## Introduction

Idiopathic pulmonary fibrosis (IPF) is a chronic and progressive disease that leads to the formation of lung scarring. The pathogenesis of IPF involves complex interactions between various cell types and signaling pathways, and the precise triggers and exact cause of IPF are still unknown. However, studies have reported that the development of IPF begins with alveolar epithelial injury in the context of predisposing factors, such as genetics, aging, environment, epigenetics, immune response, and comorbidities. Persistent injury leads to metabolic dysfunction, senescence, abnormal epithelial cell activation, and impaired epithelial repair in alveolar epithelial cells (AECs). Dysregulated AECs interact with mesenchymal cells, immune cells, and endothelial cells through multiple signaling mechanisms [1]. Molecular abnormalities involved in a series of profibrotic cellular interactions have been identified; the affected factors include reactive oxygen species (ROS), inflammatory cytokines, pulmonary surfactants, matrix remodeling factors, growth factors, and noncoding RNAs. Various cellular processes are also thought to promote lung fibrosis; such processes include cell apoptosis, oxidative stress, mitochondrial dysfunction, and endoplasmic reticulum stress. These complex changes occur as a result of AEC injury, ultimately leading to the transformation of fibroblasts into myofibroblasts, excessive deposition of extracellular matrix (ECM), pulmonary interstitial fibrosis, progressive worsening of the disease, and eventually respiratory failure and death. Current treatment options for IPF have limited efficacy. Although two drugs, pirfenidone and nintedanib, approved by the Food and Drug Administration (FDA), have been reported to delay the decline in lung function in some IPF patients, the prognosis of IPF remains poor. The median survival of newly diagnosed adult IPF patients (typically over 60 years old) is less than 5 years [2]. Lung transplantation is an effective treatment option for patients with end-stage IPF, but it is limited to a relatively young and healthy subset of patients [3]. Therefore, a better understanding of the underlying systemic pathogenic factors and mechanisms involved in IPF is crucial for optimizing IPF management and treatment.

IPF has been demonstrated to be an age-related disease [4], and changes in body composition accompany the processes of aging and obesity. Alterations in the immune-metabolic characteristics of adipose tissue and the redistribution of fat have been identified as risk factors for various age-related diseases [5]. Fat tissue not only functions to regulate temperature and store energy, as recent findings have also revealed its active role as an endocrine and immune organ. Adipose-derived factors and immune cell populations within adipose tissue impact systemic immunity and metabolism. Different immune cell populations exist in adipose tissue, and their composition and immune responses vary based on nutritional and environmental conditions. Specifically, factors such as aging and obesity promote low-grade sterile inflammation within adipose tissue and excessive infiltration of immune cells. This is accompanied by a decline in the ability of adipose tissue to store lipids, leading to ectopic fat deposition (EFD). However, cold exposure resolves obesity-induced chronic inflammation [6]. Compared to subcutaneous fat, visceral adipose tissue (VAT) is more strongly associated with chronic inflammatory diseases such as coronary artery disease, nonalcoholic steatohepatitis, diabetes, and obesity. In fact, there is also increasing recognition of the relationship between VAT and various lung diseases, including IPF. The effects of excessive VAT on pulmonary diseases include its mechanical effects on the respiratory tract, lipotoxicity, pro-inflammatory properties, and oxidative stress. Recent evidence suggests that VAT could be a modifiable risk factor for IPF [7]. However, body composition analysis of IPF patients is often overlooked, and there is currently no comprehensive review on the complex relationship between fat deposition and IPF.

There is growing interest in the role of lipids in regulating the process of pulmonary fibrosis. However, whether ectopic and visceral fat deposition serves as a profibrotic factor in the development of fibrosis and as a clinically intervenable factor remains largely unknown. This review emphasizes the frequently overlooked role of fat deposition in pulmonary fibrosis and summarizes abundant basic experiments and clinical trials. This is the first review to summarize lipid-lowering drugs, hypoglycemic drugs, and lipid-targeting drugs as a therapeutic approach for pulmonary fibrosis. By using bioinformatics methods, this review reveals lipid metabolism-related genes (LMRGs) associated with pulmonary fibrosis, introduces IPF assessment tools that are easily applicable in clinical practice, and offers novel intervention approaches from a new perspective to improve fat deposition-associated pulmonary fibrosis.

### Definition and causes of EFD

When adipose tissue dysfunction occurs or when the energy intake exceeds the storage capacity of subcutaneous adipose tissue (SAT), further calorie overload leads to excess lipid accumulation. Excess lipids accumulate in organs and tissues such as the liver, heart (pericardium, epicardium, and myocardium), lungs, intestines, pancreas, skeletal muscles, and blood vessels. This process is known as "EFD" [8]. One characteristic of EFD in humans is increased VAT accumulation, which is associated with abdominal obesity and is unrelated to body mass index (BMI) [8]. Obesity and aging significantly affect adipose tissue function by altering the spectrum of adipokines secreted by adipocytes, promoting adipocyte hypertrophy, changing the population and function of fibroadipogenic progenitor (FAP) cells, and increasing adipose tissue macrophage (ATM) infiltration [9]. These effects prevent SAT from proliferating and expanding to serve as a protective fat storage depot. In fact, several factors can contribute to increased fat deposition; these factors include high-fat diets, high-sugar diets, decreased physical activity, low serum albumin levels [10] (which binds and transports free fatty acids [FFAs]), male sex, and hormonal imbalance [11].

### EFD in the lung induces alveolar structural and functional damage in IPF

Accumulating evidence indicates that a high-fat diet promotes lung fibrosis [12]. In obese individuals, fat can directly accumulate in the lung and airways; adipose tissue can be found in the outer walls of the larger airways, correlating with BMI, airway wall thickness, and higher neutrophil counts [13]. Studies on obese animal models have shown elevated levels of phospholipids and triglycerides in lung tissue [14]. Abundant lipid droplets can be observed in the pulmonary interstitium and lung macrophages, concomitant with the destruction of ultrastructural features of alveolar epithelial type II cells (AT2), expansion of rough endoplasmic reticulum, reduced cellular biosynthesis, impaired secretion of lung surfactant, and increased interstitial collagen [15]. Animal studies have revealed that obese diabetic rats exhibit a 136% increase in total lung triglyceride content, a 32% increase in interstitial collagen fibers, and a reduced diffusing capacity of the lungs for carbon monoxide (DLCO) [16].

EFD can also occur in lung lipofibroblasts (LIFs) of obese individuals. LIFs are important lung stromal cells that are commonly found adjacent to AT2 cells and support the self-renewal and differentiation of AT2 stem cells to AT1 cells. LIFs provide triglycerides to AT2 cells for the synthesis of pulmonary surfactant [17]. Fat deposition associated with diabetes, obesity, and aging leads to impaired function of lung LIFs, compromising their ability to aid in the renewal of AECs and maintain alveolar lipid homeostasis. Furthermore, dysfunctional LIFs can directly transdifferentiate into myofibroblasts, resulting in excessive ECM production and subsequent pulmonary fibrosis [18–20].

### Lipotoxicity of fat deposition and IPF: direct cytotoxicity and indirect proinflammatory effects

#### Lipotoxicity of FFAs to AECs promotes pulmonary fibrosis

The profibrotic role of pulmonary EFD is associated with the lipotoxicity of excessive fatty acids on AECs. Enlarged adipocytes also exhibit enhanced lipolysis, leading to increased delivery of FFAs to other organs. Increased FFA levels can disrupt the integrity of biological membranes in EFD tissues and alter cellular acid‒base homeostasis. FFAs have been shown to activate Toll-like receptor 2 (TLR-2), TLR-4/nuclear factor-kappaB (NF-κB), and c-Jun N-terminal kinase (JNK) signaling pathways, thereby promoting inflammation and insulin resistance [21, 22]. Furthermore, FFAs serve as precursors for the synthesis of harmful bioactive lipids, particularly ceramides and diacylglycerols. Overall, the deleterious effects resulting from the secretion of adipokines, lipid molecules, and inflammatory factors from ectopic fat tissues are referred to as "lipotoxicity."

Elevated levels of palmitic acid esters (a saturated FFA) have been observed in the lungs of patients with IPF, leading to endoplasmic reticulum stress and apoptosis in AECs. This phenomenon has been confirmed in a bleomycin (BLM)-induced IPF mouse model fed different diets [23]. The lipotoxicity of AECs induced by a high-fat diet suggests that EFD contributes to the initiation of IPF and exacerbates fibrosis severity. In addition to inducing endoplasmic reticulum stress and AEC apoptosis, lung EFD has been associated with increased lipid levels in bronchoalveolar lavage fluid (BALF) in a BLM-induced model. Alveolar macrophages engulf extracellular oxidized phospholipids and transform into lipid-laden foam cells, releasing more transforming growth factor beta1 (TGF-β1) and further exacerbating pulmonary fibrosis [24]. Lipid-lowering agents and cluster of differentiation 36 (CD36, a fatty acid translocase) inhibitors or CD36 gene knockout reduced the differentiation of lung fibroblasts to myofibroblasts in BLM mice [25, 26]. This suggests that EFD plays a crucial role in pulmonary fibrosis through macrophage-CD36 oxidative lipid signaling.

Further metabolites of FFAs, known as bioactive sphingolipids, such as sphingosine-1-phosphate (S1P), play an important role in the pathogenesis of pulmonary fibrosis [27]. Under conditions of nutrient overload, S1P synthesis increases using neural-derived sphingolipids as substrates, and S1P acts as a second messenger by autocrine or paracrine binding to G protein-coupled receptors. Studies have shown that the levels of sphingosine kinase 1 (SPHK1, catalyzing the generation of S1P) are significantly increased in IPF patient lung tissues and strongly correlated with α-smooth muscle actin (α-SMA), vimentin, and type I collagen. S1P and SPHK1 levels in BALF, serum, and peripheral blood monocytes of IPF patients are negatively correlated with lung function and positively correlated with mortality rate [28]. Animal and cell experiments have shown that the SPHK1/S1P signaling pathway is associated with TGF-β signaling, promoting the activation of fibroblasts and their transformation into myofibroblasts through the activation of mitochondrial Rho kinase, the Hippo/YAP (Yes-associated protein) pathway, etc. [29–31].

#### Mechanism of adipose-derived adipokines in pulmonary fibrosis

In addition to lipid molecules such as FFAs, adipose-derived adipokines are also considered key participants in the development of pulmonary fibrosis in IPF patients and BLM-treated mice. Changes in the secretion levels of various adipokines, including hormones (such as leptin and adiponectin) and peptides (such as angiotensinogen, apelin, resistin, and plasminogen activator inhibitor-1 [PAI-1]), have been observed in obese and elderly patients [32, 33]. Leptin and adiponectin play a role in the pathogenesis of obesity-related lung diseases by affecting systemic inflammation, regulatory T (Treg) cell activity, and T helper cell 17 (Th17) and T helper cell 2 (Th2) immune responses [34]. It is known that aging, a high-fat diet, and adipose tissue dysfunction caused by obesity increase the leptin/adiponectin ratio, which is associated with lung function and fibrosis markers [35]. Serum leptin levels are positively correlated with body fat and negatively correlated with lung function. In contrast to leptin, adiponectin levels are decreased in subjects with impaired lung function and obesity [36].

Leptin is secreted by adipocytes in white adipose tissue, and leptin receptors are highly expressed on the surface of alveolar macrophages. The binding of leptin to its receptor drives the activation of the NOD (nucleotide oligomerization domain)-like receptor thermal protein domain associated protein 3 (NLRP3) inflammasome. This leads to the production of pro-inflammatory and pro-fibrotic cytokines, such as interleukin (IL)-1, IL-18, and TGF-β, promoting AEC mitochondrial stress, cellular apoptosis, and insulin resistance [37]. Activation of the NLRP3 inflammasome is also closely associated with increased collagen deposition and enhanced expression of connective tissue growth factor in pulmonary fibrosis [38]. Increased IL-1β signaling in the lungs promotes the expression of proinflammatory cytokines (such as IL-23 and IL-5) and recruits T cells, B cells, and eosinophils to produce IL-13 and TGF-β1, which are critical regulatory factors for fibroblast activation and excessive ECM production [39]. However, VAT has a stronger negative correlation with adiponectin than subcutaneous fat [40]. Adiponectin was identified as an initiator of AMP-activated protein kinase (AMPK)-dependent autophagy.

Deficiency of adiponectin, which is associated with EFD, can lead to the generation of ROS and potassium efflux. This induces mitochondrial dysfunction and results in lung injury and activation of the NLRP3 inflammasome [41]. Adiponectin has also been identified as an anti-atherosclerotic, anti-inflammatory, and anti-diabetic adipokine, and these protective effects are attributed to its impact on the activation of the NF-kB (nuclear factor kappa B) pathway in B cells, which enhances insulin sensitivity [37, 42].

Another important adipokine is angiotensinogen (AGT), which is produced by adipose tissue and accounts for one-third of the circulating AGT levels. In the obese state, adipose tissue-produced AGT increases [43], leading to excessive activation of the local adipose tissue and systemic renin-angiotensin system (RAS) [44–46]. Studies have revealed that patients with the ID/DD (insertion/deletion) polymorphism of angiotensin-converting enzyme (indicating higher levels of the enzyme) are prone to pulmonary fibrosis [47]. Angiotensin II (Ang II) has been identified as a pro-apoptotic and pro-fibrotic factor in experimental pulmonary fibrosis animal models. In human lung fibroblast cultures, Ang II induces the activation of TGF-β1/Smad2/3, promoting fibroblast-myofibroblast transition [48]. Elevated Ang II levels in the local or circulation of mouse lungs can induce progressive pulmonary fibrosis, while renin inhibitors such as aliskiren or angiotensin II type 1 receptor-specific antagonists, such as losartan, can block the production of ECM proteins and fibrogenic factors [49, 50].

**Fig. 1:**
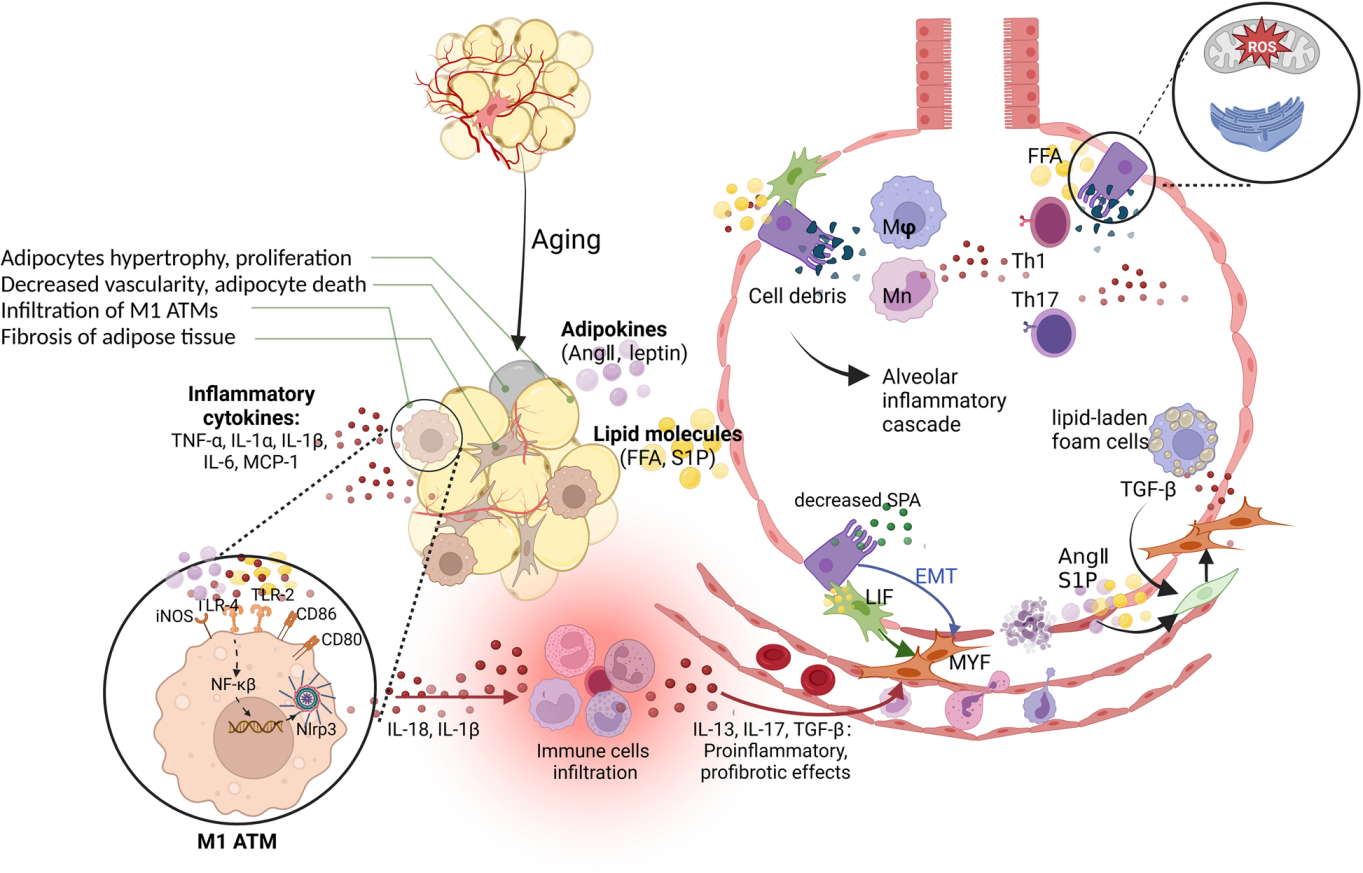
Alterations in aging adipose tissue and the involvement of fat deposition in the occurrence and development of IPF. 1) During the aging process, excessive expansion of adipose tissue leads to hypoxia. This stimulates adipocytes and ATMs to secrete inflammatory chemokines, resulting in immune cell infiltration in aging adipose tissue. 2) Fibrosis in dysfunctional adipose tissue leads to lipotoxicity and an increased leptin/adiponectin ratio. This activates highly proinflammatory M1-type macrophages (M1 ATMs) through molecules such as leptin, PAI-1, FFA, and inflammatory cytokines, thereby exacerbating the inflammatory response. 3) Lipotoxicity and inflammation in aging adipose tissue leads to endoplasmic reticulum stress, mitochondrial dysfunction, apoptosis, autophagy and necrosis of AT2 cells. Subsequently, in the alveoli, cell debris, recruited immune cells, and foam cells (macrophages engulfing lipid droplets) participate in the inflammatory cascade response, resulting in fibroblast-to-myofibroblast (MYF) transformation and epithelial-mesenchymal transition (EMT). 4) Adipose factors such as Ang II, PAI-1, and S1P can also promote fibroblast-to-MYF transformation. 5) Lipotoxicity and inflammation not only promote the differentiation of LIFs into MYFs but also affect the supply of pulmonary surfactant precursors to AT2 cells. The figure was created using BioRender (www.biorender.com). Abbreviations: adipose tissue macrophages (ATMs), plasminogen activator inhibitor-1 (PAI-1), free fatty acids (FFA), alveolar epithelial type II cells (AT2), myofibroblast (MYF), epithelial-mesenchymal transition (EMT), Angiotensin II (Ang II), sphingosine-1-phosphate (S1P), lipofibroblasts (LIFs)

Similar to Ang II, the adipokine PAI-1 is also overexpressed and released by adipocytes in obesity; it has been shown to have a definite promoting effect on pulmonary fibrosis [51]. PAI-1 is a recognized inhibitor of fibrinolysis and can also affect the functionality of fibronectin, thereby interfering with cell adhesion [52]. Its overexpression contributes to the accumulation of ECM. PAI-1 is increased in the lungs of patients with pulmonary fibrosis. It not only promotes fibrosis but also activates alveolar macrophages to promote inflammation, and through TGF-β1, it strongly induces AT2 cell senescence [53]. However, it should be noted that the current research on the direct relationship among Ang II, PAI-1 sourced from excessive adipose tissues, and IPF in humans is still limited in terms of quantity. Considering that visceral fat is one of the main sources of fibrotic and inflammatory factors, further research into the mechanisms underlying the association between visceral fat and fibrosis is crucial. The changes in aging adipose tissue and the involvement of fat deposition in the occurrence and development of IPF are shown in Fig. 1.

### Insulin resistance and immune cell infiltration in the fat deposition of lungs promote IPF

#### Insulin resistance in fat deposition promotes IPF through TGF-β signaling

Insulin resistance caused by elevated levels of adipokines, resistin and retinol-binding protein 4 and reduced levels of adiponectin is another potential mechanism for the occurrence and development of IPF [54]. Additionally, enlarged fat cells release proinflammatory cytokines, including tumor necrosis factor-alpha (TNF-α), IL-6, IL-8, and monocyte chemotactic protein-1 (MCP-1), leading to serine phosphorylation of insulin receptor substrate-1 (IRS-1) production and blocking insulin signal transduction. This consequently reduces insulin sensitivity and causes insulin resistance, which is a key feature of metabolic syndrome [55]. Compared to elderly patients without metabolic syndrome, elderly patients with metabolic syndrome have higher airway resistance. They also exhibit higher levels of proinflammatory mediators, such as leptin, IL-1β, IL-8, and TNF-α, lower levels of anti-inflammatory mediators, including adiponectin, IL-1 receptor antagonist, and IL-10, and increased expression levels of TGF-β1 and phosphorylated Smad-2/3 [35]. In mice, intranasal insulin administration enhances bronchial epithelial TGF-β1 expression, activating the TGF-β/Smad signaling pathway and causing fibrosis around the airways and blood vessels. TGF-β also stimulates the differentiation of Th0 cells into Th17 cells, which release IL-17 and contribute to airway hyperreactivity [54]. Serum vitamin D and NAD (nicotinamide adenine dinucleotide)-dependent deacetylase sirtuin (SIRT), an anti-aging factor, levels are decreased under conditions of insulin resistance. Vitamin D deficiency inhibits the phosphorylation of Smad-2/3, activates RAS activity, and subsequently activates TGF-β1 signaling, promoting pulmonary fibrosis [56]. SIRT-1 has been shown to inhibit NF-κB activity and reduce inflammation through various mechanisms, including inhibiting iNOS (inducible nitric oxide synthase) activity and downregulating COX-2 (Cyclooxygenase-2) expression, thereby alleviating oxidative stress. Aerobic exercise in obese mice improves insulin resistance, reduces neutrophil infiltration in the lungs, decreases pro-inflammatory, pro-oxidative stress, and pro-fibrotic factors in BALF, and upregulates the expression of anti-inflammatory factors IL-10 and SIRT-1 mRNA in the lungs [57]. Furthermore, studies have indicated that SIRT-1 acts as a target for anti-pulmonary fibrosis drugs and inhibits the EMT in BLM-induced pulmonary fibrosis in mice [58].

#### Fat deposition participates in the pathogenesis of IPF through immune cell infiltration

The presence of inflammatory cells in dysfunctional adipose tissue can affect adjacent tissues and organs [59]. As mentioned earlier, ectopic fat can be directly deposited in airways, alveolar interstitium, lung LIFs, and alveolar macrophages, indicating that the lungs can be directly influenced by inflammatory factors released from local adipose tissue and immune cell infiltration. Enlarged adipocytes and reduced capillary density in hypertrophic adipose tissue lead to a hypoxic state in adipocytes, characterized by abnormal preadipocyte differentiation, inflammation, altered secretion profile, increased oxidative stress and mitochondrial dysfunction in adipocytes, and accumulation of aged fat cells and fibrosis in adipose tissue [60]. The differentiation of preadipocytes to adipocytes is decreased, and instead, their differentiation to ATMs expressing surface markers, such as F4/80, CD80, and CD86, is increased. Moreover, adipocytes undergo hypoxic cell death, recruiting a large number of monocytes through MCP-1. These monocytes differentiate into proinflammatory M1 macrophages and form “crown-like structures,” a process activated through the NLRP3 pathway [61]. During the formation of crown-like structures, lipid metabolism increases in ATMs, leading to lipotoxicity, inflammation, and enhanced insulin resistance [62].

In obese and elderly VAT, ATMs are the most abundant immune cells. These cells account for 10% of immune cells in normal subjects and 50% of immune cells in obese individuals, and the ratio of M1 ATMs (proinflammatory characteristics) to M2 ATMs (anti-inflammatory characteristics) is significantly increased in obese individuals [63]. Hypoxia may induce inflammation through hypoxia-inducible factor 1-alpha (HIF-1α) gene expression, triggering the secretion of proinflammatory mediators such as TNF-α, IL-6, IL-8, MCP-1, adipokines, and retinol-binding protein by hypertrophic adipocytes and M1 ATMs and promoting further immune cell infiltration [64, 65]. Lymphocytes constitute the second most abundant immune cell population in the VAT of obese and elderly patients. There was a twofold increase in CD3 + T cells, predominantly CD8 + T cells (cytotoxic T cells), in aged mouse VAT compared to young animal VAT, and a similar trend was observed in obese mice [66, 67]. NLRP3 regulates IL-18 and interferon-γ (IFN-γ) in white adipose tissue and promotes the differentiation of effector CD8 + T cells, releasing proinflammatory and profibrotic molecules, such as IL-13 and IL-17, and M1 ATMs and alveolar macrophages. This leads to lung and systemic inflammation and insulin resistance [68]. Previous studies have shown a significant increase in the expression levels of IL-1β, IL-8, and IL-6 in BALF and lung tissue of pulmonary fibrosis patients and in animal models, and IL-1β or IL-6/IL-13 activation of JAK2 (Janus kinase 2) and STAT3 (Signal transducer and activator of transcription 3) stimulates primary AT2 and lung fibroblasts. This stimulates the release of TGF-β1 by immune cells and fibroblasts, which induces EMT and fibroblast-to-MYF transformation, and promotes AT2 cell aging and an apoptotic phenotype [69, 70]. In the BLM-induced lung fibrosis animal model, lung inflammation, fibrosis, and collagen deposition depend on the IL-1R1/MyD88 signaling pathway [71]. Elevated levels of IL-6 (> 25.20 pg/mL) are an independent risk factor for acute exacerbation (AE-IPF) (odds ratio [OR] 1.014, *p* = 0.036) and mortality (OR 1.007, *p* = 0.018) in patients with interstitial lung diseases [72]. IL-17A inhibits autophagy in bronchial epithelial cells through the PI3K/Akt/mTOR pathway [73]. It also promotes lung fibroblast proliferation and contributes to lung inflammation and fibrosis through the IL-17A-TGFβ axis. The primary function of IL-8 is to amplify the differentiation of mesenchymal stem cells to fibroblasts, promote lung fibroblast proliferation and migration, recruit and activate macrophages, and play a crucial role in airway fibrosis and remodeling [74].

In recent years, it has been demonstrated that ectopic adipose tissue outside the lungs is also involved in the pathogenesis of IPF. Excessive pericardial adipose tissue is a rich source of proinflammatory mediators in the systemic circulation and has been associated with higher levels of inflammatory markers (IL-6, TNF-α, MCP-1, CD11c, and iNOS) and fibrotic markers (collagen levels, TGF-β, matrix metalloproteinase-3) in various cardiovascular and pulmonary diseases, such as COVID-19 (Coronavirus Disease 2019), COPD, pulmonary arterial hypertension, sleep apnea syndrome, heart failure, coronary heart disease, and lung transplant recipients. Therefore, excessive pericardial adipose tissue indicates a poor prognosis of these diseases. In 2021, Anderson MR and his colleagues found that for each doubling in pericardial adipose tissue volume, the odds of interstitial lung abnormalities increased by 20%, while the FVC (forced vital capacity) percentage predicted a decreased of 5.5%. The study also identified the involvement of IL-6 and leptin in the association between adipose tissue and lung fibrosis [75]. These findings suggest that proinflammatory cytokines and adipokines from ectopic adipose tissue outside the lungs can enter the pulmonary circulation and cause lung injury. In addition to inflammatory factors and adipokines, the neutrophil-to-lymphocyte ratio (NLR) and serum hs-CRP levels have also demonstrated a positive correlation with pericardial adipose tissue volume, and a high NLR has been shown to independently influence the occurrence of IPF [76, 77].

### Fat deposition aggravates lung function loss in IPF

#### Mechanism of sarcopenia caused by fat infiltration

Studies suggest that fat deposition aggravates lung function loss in IPF, including but not limited to increased fat infiltration in skeletal muscle, airway and pericardial adipose tissues. Skeletal muscle fat infiltration and skeletal muscle atrophy are considered to be harmful to muscle mass, strength, activity, and muscle metabolism [78]. The most common cause of death in IPF patients is chronic respiratory failure, and skeletal muscle atrophy and skeletal muscle fat deposition are very common in patients with respiratory failure requiring mechanical ventilation and malnutrition [79]. Additionally, these factors have been shown to increase the risks of hospitalization and death in IPF [80]. Chun-wei Li et al. proposed that dysfunction of adipocytes caused by aging and obesity is the earliest driving factor of local inflammation and insulin resistance [81]. This is followed by a systemically expanded vicious loop called “the metabaging cycle,” in which excessive lipids can “spill over” into skeletal muscle tissue. These lipids accumulate in the form of intermuscular lipids, intramyocellular lipids, and lipid droplets within muscle cells, leading to the accumulation of toxic lipids such as diacylglycerol and ceramides in skeletal muscle tissue [81]. Ceramides directly induce insulin resistance in skeletal muscle cells by blocking downstream signaling of insulin, such as the translocation of glucose transporter-4 (the main glucose transporter for glucose uptake in skeletal muscle) [82]. Various other obesity-related lipid metabolites, such as homocysteine, free fatty acids, ROS, uric acid, and cholesterol crystals, activate the NLRP3 inflammasome to induce the production of IL-1β and IL-18 by macrophages. These cytokines can then further promote inflammation in T cells, impairing skeletal muscle insulin sensitivity [83]. Muscle tissue is one of the primary effectors of insulin. Insulin resistance in muscle leads to restricted glucose uptake and synthesis of muscle glycogen, as well as limited lipid uptake by muscle tissue. As a result, blood glucose is directed toward the synthesis of more fat in adipocytes, leading to the further elevation of free fatty acid concentrations and local hyperlipidemia. The increased blood glucose load contributes to systemic endogenous free radicals and inflammation, perpetuating the metabaging cycle [84].

In obesity, factors such as TNF-α, IL-18, IL-6, and iNOS are released by M1 ATMs, leading to reactive atrophy of skeletal muscle tissue and a decrease in the number of muscle cells [85]. As a population of mesenchymal stem cells, FAPs (fibro-adipogenic progenitors) possess multipotent differentiation potential, including the ability to differentiate to fibroblasts, adipocytes, chondrocytes, and osteoblasts [86]. When regulated by paracrine signals from adipose tissue proinflammatory factors, FAPs in skeletal muscle can differentiate to a fat cell-like phenotype, leading to reduced muscle cell regeneration and increased skeletal muscle fat infiltration. TNF-α, released by M1 ATM1s, plays a crucial role in the process of muscle wasting and fat infiltration within skeletal muscle. Studies have shown that high levels of TNF-α directly impair mitochondrial biogenesis in muscle cells and disrupt myotube formation in human primary myoblasts [87]. Additionally, TNF-α, through the activation of TNF receptor 1, triggers the activation of the caspase cascade, increasing apoptosis of muscle cells and FAPs. This subsequently increases the release of TNF-α and exacerbates the vicious cycle. TNF-α not only induces programmed cell death in skeletal muscle cells but also upregulates ROS directly or indirectly through adipocyte necrosis and lipotoxicity. This, in turn, activates the NF-κB pathway and upregulates the expression of muscle-specific E3 ubiquitin ligase, muscle RING-finger protein-1 (MuRF1), promoting proteolysis of myofibrillar proteins and muscle wasting [88]. In summary, the deposition of intramuscular lipids demonstrates significant lipotoxicity, leading to the induction and aggravation of mitochondrial dysfunction, oxidative stress, insulin resistance, and inflammation. These molecular changes interact with each other, resulting in a vicious cycle that impairs muscle regeneration and ultimately increases the risk of systemic muscle wasting or cachexia [51].

#### Muscle fat infiltration is associated with lung function loss

The mechanisms underlying muscle wasting due to EFD can explain the prognostic differences observed in different nutritional phenotypes in IPF patients. In a prospective study of 90 IPF patients, the proportions of normally nourished, nonsarcopenic obese, sarcopenic and sarcopenic obese (muscle loss with increased visceral fat) patients were 67.8%, 25.3%, 4.6%, and 2.3%, respectively [89]. Compared to patients with nonsarcopenic obesity or sarcopenia, patients with sarcopenic obesity showed decreased protein synthesis and increased protein breakdown in respiratory muscles. These patients also exhibited a reduction in respiratory muscle mitochondria and mitochondrial dysfunction compared to healthy control individuals [90]. This suggests a synergistic amplification of adverse consequences through the metabaging cycle formed by increased EFD and skeletal muscle loss, leading to maximized metabolic damage, decreased quality of life, and increased morbidity and mortality rates of IPF. IPF is a restrictive lung disease, and there is strong evidence from large-sample studies suggesting that sarcopenic obesity is primarily associated with an increased risk of restrictive lung disease in the elderly (OR 2.81, 95% confidence interval [CI]: 1.72–4.59). The sarcopenic obesity group had a significantly lower FVC than the normal control group, while the FEV1/FVC ratio (an indicator of obstructive ventilation) was not significantly different between the two groups [91]. The distribution of visceral fat and changes in muscle mass also explain the contradictory observations of BMI in the prognosis of IPF. Evidence suggests that weight loss in IPF indicates an increased risk of hospitalization and worse prognosis [92, 93]. However, some studies have also revealed a protective effect of high BMI on survival in respiratory disease patients [94]. This “obesity paradox” is partly due to the limitations of using BMI to measure visceral obesity [95]. This suggests that weight, BMI, or other body composition indicators may not be suitable prognostic indicators for IPF, and more direct measures of body composition need to be determined. Quantification of skeletal muscle, visceral fat, and lean body mass has become a new hotspot in research [96, 97]. Studies have demonstrated that sarcopenia (decreased quantity and poor physical performance) in patients with IPF is associated with high severity, poor quality of life and poor prognosis [98–103]. Inspiratory muscle training in IPF patients who can tolerate pulmonary rehabilitation is beneficial because it partially offsets muscle fat infiltration and muscle mass reduction associated with aging and improves disuse muscle atrophy [104, 105].

In conclusion, the damage inflicted by muscle fat deposition in IPF patients is multifactorial, including its impact on respiratory muscle dysfunction contributing to respiratory failure, systemic inflammation, oxidative stress, and cachexia. These findings may have substantial implications for the management of IPF patients, and the assessment of body composition, including muscle and visceral fat, should become a routine clinical practice in IPF. Future research can evaluate nutritional interventions based on patients' nutritional phenotypes and develop personalized respiratory muscle training and other pulmonary rehabilitation programs.

#### Other factors lead to a negative effect on lung function

In addition to sarcopenia and respiratory weakness caused by respiratory muscle fat infiltration, there are at least three other factors that contribute to the negative impact of fat deposition on lung function in IPF patients. 1) Fat deposition in the visceral cavity produces mechanical obstructive effects on the respiratory tract and restrictive effects on the diaphragm. 2) Lipotoxicity resulting from fat deposition damages alveolar ultrastructure, reduces surfactant production, and promotes lung tissue fibrosis, leading to pulmonary diffusion dysfunction. It also leads to mild systemic inflammation that impairs lung immune responses and increases airway hyperresponsiveness (as discussed in Sects. " EFD in the lung induces alveolar structural and functional damage in IPF" and " Lipotoxicity of fat deposition and IPF: direct cytotoxicity and indirect proinflammatory effects" of this review). 3) Fat deposition is involved in various IPF complications, including OSAS, pulmonary hypertension, COPD, and hemodynamic disturbance caused by increased pericardial fat [106]. EFD in the mediastinum and abdominal cavity limits lung expansion, leading to a significant decrease in expiratory reserve volume and functional residual capacity. The reduction in functional residual capacity is directly proportional to the severity of obesity, with overweight, mildly obese, and severely obese subjects presenting reduction rates of 10%, 22%, and 33%, respectively [107]. Fat deposition in the airways, extrapleural space, and chest wall reduces lung compliance and increases respiratory resistance, resulting in a direct mechanical impact on respiratory function. Chronic lipotoxicity primarily affects lung diffusing capacity, while the cardiopulmonary complications of IPF mainly lead to ventilation/perfusion (V/Q) mismatch. Compared to patients with other chronic lung diseases, IPF patients often experience more common hypoxemia and accompanying pulmonary hypertension due to impaired V/Q balance, which also limits tolerance to pulmonary rehabilitation therapy in IPF. The one-year incidence of AE-IPF is approximately 16.5%, and EFD-related IL-6 and IL-8 are predictive factors for the early onset of AE-IPF [108]. The pulmonary function impairment caused by EFD is of considerable importance in lethal AE-IPF cases [109]. In multivariate analysis, resting hypoxemia requiring oxygen therapy ([hazard ratio]HR 2.44, 95% CI: 1.45–4.10), every 10% decrease in FVC percentage predicted (HR 1.28, 95% CI: 1.10–1.49), and every 10% decrease in DLCO percentage predicted (HR 1.25, 95% CI: 1.04–1.51) were significantly associated with an increased risk of death or lung transplantation in IPF patients [110].

It has been shown that obesity-induced impaired lung function in patients can be effectively reversed through weight loss surgery [111]. In experimental animals that underwent gastric sleeve surgery, an improvement in alveolar structure, a reduction in collagen fiber and lipid deposition, an inhibition of the excessive proliferation of chronic hypoxia-induced capillary basement membrane, and an increase in capillary blood supply were observed [15]. Fortunately, fat deposition and lung function impairment caused by aging can be partially improved through dasatinib and quercetin. Senolytics are a class of drugs that selectively induce the death of senescent cells [5], and dasatinib and quercetin constitute the first combination of senolytic drugs. Dasatinib can eliminate aged adipocyte progenitor cells [112] and reduce the secretion of inflammatory mediators in aging VAT. With senolytics treatment, the BLM-induced lung fibrosis mice showed downregulation of the inflammatory pathway in lung tissue and significant improvements in lung function and physical fitness [2]. In the first human trial, treatment with dasatinib and quercetin resulted in an average improvement of 21.5 m in the 6-min walking distance of elderly patients [113]. With the progress of preclinical and phase I clinical trials, senolytics have shown great therapeutic prospects in IPF [114].

### Fat deposition promotes complications in IPF, intensifies the pathogenicity of environmental factors in IPF, and aggravates IPF prognosis and lung transplant outcomes

#### Fat deposition contributes to complications of IPF

The majority of IPF patients have pulmonary and/or extrapulmonary complications. Only 60–70% of deaths are directly attributable to IPF-related conditions, with the cause of death in the remaining patients likely being other comorbidities present in the elderly population [115]. In a meta-analysis that included 126 studies, pulmonary complications in IPF included pulmonary hypertension (prevalence rate 3–86%), COPD (6–67%), OSAS (6–91%), and lung cancer (3–48%), and the nonpulmonary diseases included type 2 diabetes (10–42%), ischemic heart disease (3–68%), congestive heart failure, gastroesophageal reflux disease (0–94%), sarcopenia, anxiety and/or depression [116]. These complications have been shown to be related to the functional status, quality of life, and survival time of IPF. This is particularly true for lung cancer and pulmonary hypertension, which have the most substantial impact on the survival and lung transplant outcomes of IPF patients [117, 118]. Additionally, the cumulative number of complications is negatively correlated with IPF survival rates. Pulmonary hypertension before transplantation is associated with poor posttransplant survival [HR 4.832, *p* = 0.039] and increases the risk of posttransplant complications [119]. The EMPIRE registry study included 3,580 IPF patients from multiple countries, and at the time of enrollment, 91.3% of patients had been diagnosed with at least one comorbidity, with over one-third (37.8%) reporting four or more comorbidities. The 5-year survival rates for patients without common complications and with 1, 2, 3, and ≥ 4 complications were 53.7%, 48.4%, 47.0%, 43.8%, and 41.1%, respectively [120].

#### Fat deposition increases the risk of complications in IPF

**Fig. 2:**
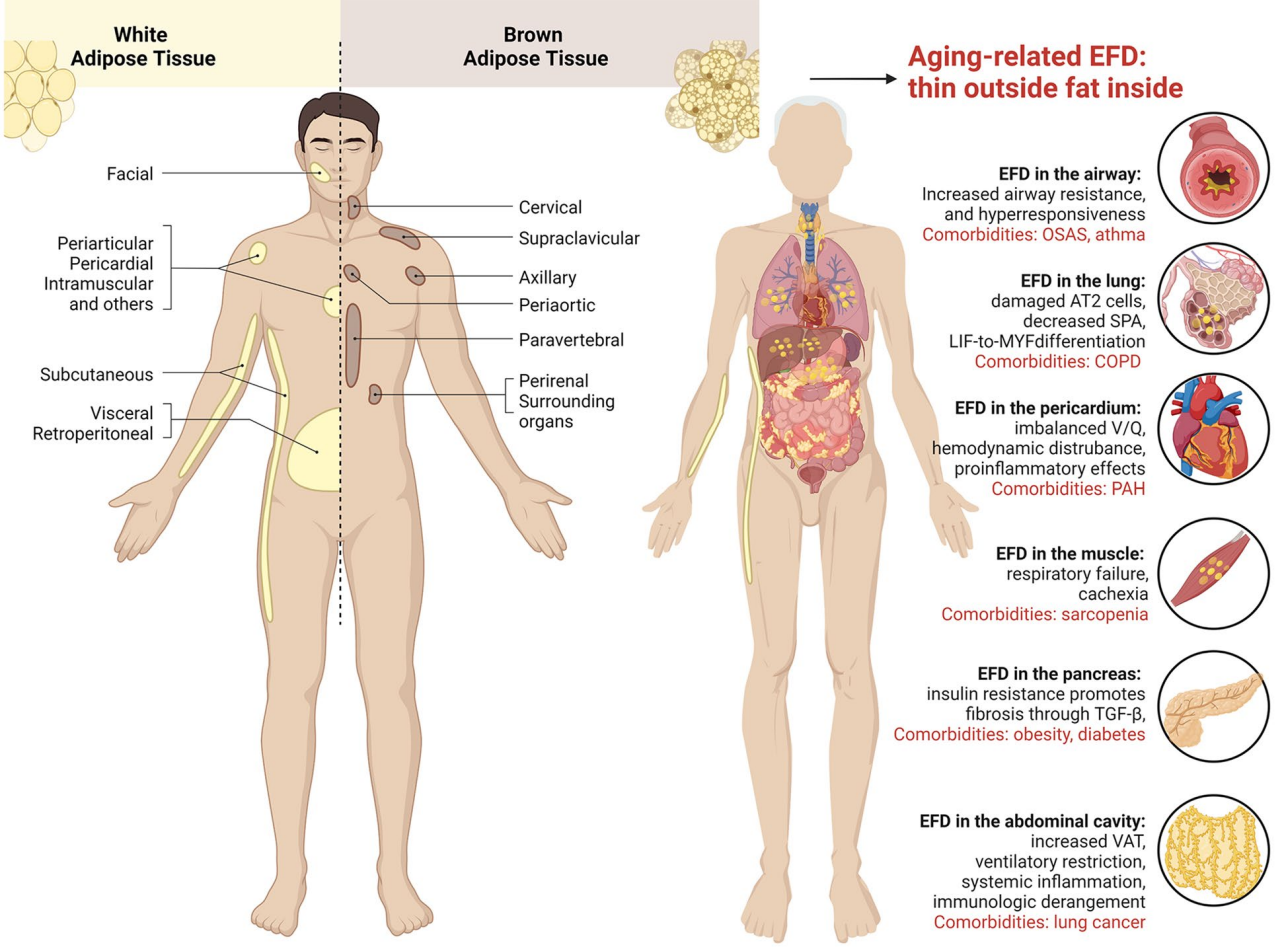
Alterations in adipose tissue distribution in aging individuals contribute to the development of IPF. The left part illustrates the distribution of white adipose tissue and brown adipose tissue in the healthy human body. The right part shows a list of comorbidities associated with an excessive accumulation of ectopic fat and visceral adipose tissue in elderly individuals. The figure was created using BioRender (www.biorender.com). Abbreviations: alveolar epithelial type II cells (AT2), chronic obstructive pulmonary disease (COPD), insulin resistance (IR), lipofibroblast (LIF), myofibroblast (MYF), obstructive sleep apnea syndrome (OSAS), pulmonary arterial hypertension (PAH), surfactant protein A (SPA)

These comorbidities share common risk factors with IPF, and one of these factors is fat deposition (Fig. 2). Furthermore, fat deposition exacerbates the pathogenicity of environmental factors (such as exposure to cigarette smoke and pathogens) on IPF and its comorbidities. For instance, fat deposition not only plays a role in the development of IPF through mechanisms such as lipotoxicity, inflammation, oxidative stress, and fibrogenesis but also has direct evidence of fat deposition in the pancreas, leading to pancreatic fat infiltration. This pancreatic fat infiltration contributes to the occurrence of diabetes, which is a common pulmonary comorbidity in IPF patients [121]. Adipocytes mainly infiltrate the pancreatic parenchyma and accumulate near islets. The number of D68-positive cells in islets is positively correlated with homeostatic model assessment of insulin resistance (HOMA-IR) and the area of pancreatic adipocytes and leads to intensified local inflammation, β-cell apoptosis promotion, and alterations to insulin secretion and glucose tolerance [122], which are well-known mechanisms of diabetes. A case‒control study showed that type 2 diabetes is an independent risk factor for IPF, with a higher incidence of diabetes in IPF patients than in patients without IPF (11.3% vs. 2.9%) [119, 123]. A meta-analysis of 260,000 individuals revealed that the odds of having diabetes were increased by 1.54 times in IPF patients compared to patients without IPF (95% CI, 1.30–1.84; *P* < 0.001) [124]. Another meta-analysis of nine case‒control studies also reported similar results (OR 1.65, *P* < 0.0001) [125]. In a cohort study, the presence of diabetes (HR 2.5, 95% CI 1.04–5.9) was identified to increase mortality in the IPF cohort [126]. Based on this evidence, the co-occurrence and connection of pancreatic and pulmonary pathologies in IPF can be partially explained by EFD. In addition to extrapulmonary comorbidities, EFD is also involved in respiratory system comorbidities in IPF. The mechanical effects of EFD on airway caliber, lung capacity, and cardiac diastole are mainly associated with COPD, OSA, and pulmonary hypertension, while its promotion of inflammation and airway hyperresponsiveness is mainly associated with asthma and increased pathogenicity of environmental factors (such as COPD and pulmonary infections) [127, 128]. Fat deposition leads to decreased numbers and functional defects of natural killer cells, resulting in impaired malignant cell clearance and an increased risk of lung cancer [129]. Its impact on respiratory muscle depletion is mainly related to respiratory failure and cachexia in COPD and lung cancer [130]. Long-term hypoxemia contributes to the occurrence of pulmonary heart disease.

### Drugs of hypoglycemic or lipid-lowering and targeted lipid-mediated pathways for pulmonary fibrosis

An increasing number of researchers believe that hyperglycemia and lipid deposition may be risk factors for pulmonary fibrosis, which is closely associated with systemic inflammation and oxidative stress. In recent years, various drugs for glycemic regulation and lipid modulation have shown antifibrotic properties. Among them, hypoglycemic drugs, including empagliflozin (a sodium-glucose cotransporter-2 inhibitor), liraglutide (a glucagon-like peptide 1 receptor agonist), metformin, and rosiglitazone, have been shown to have good effects in alleviating pulmonary fibrosis in various animal models (see Table 1). Lipid-lowering drugs have also attracted attention, and studies have shown that fenofibrate, pravastatin, atorvastatin, ezetimibe and probucol can significantly reduce the development of pulmonary fibrosis in animal models (see Table 1, Ref. [131–139]).

**Table 1:** Animal experiments of representative hypoglycemic or lipid-lowering drugs for pulmonary fibrosis

Drug	Mechanism	Model	Control Group	Experimental Group	Ref
Empagliflozin	Modulating Sesn2/AMPK/Nrf2 signaling Targeting ferroptosis and autophagy	BLM-induced PF in rats (BLM, I.P.)	Saline (I.P.) and 1% CMC (I.G.) BLM (I.P.) EMPA dissolved in 1% CMC (I.G.)	EMPA (I.G.) for 7d before BLM and continue for 4w after BLM (I.P.)	[131]
Liraglutide	Reducing collagen interstitial deposition and production of precursor materials Reducing myofibroblasts in the tissues and the expression of pro-fibrotic cytokines	BLM-induced PF in rats (Intratracheal instillation of BLM)	VEH (S.C.) after intratracheal instillation of BLM	Liraglutide dissolved on 0.4% acetic acid (S.C.) after intratracheal instillation of BLM	[132]
Metformin	Targeting S100A4 via AMPK-STAT3 axis	BLM-induced PF in mice (Intratracheal instillation of BLM)	Intratracheal instillation of Saline Intratracheal instillation of BLM	MET (I.P.) (Day 7 after treatment of BLM or saline)	[133]
Rosiglitazone	Upregulating PTEN and downregulating the TGF-β1 expression in a PPAR-γ dependent manner	PQ-induced PF in rats (PQ, I.P.)	Distilled water (I.P.) RSG (I.P.) GW (I.P.)	PQ (I.P.) PQ + RSG (I.P.) PQ + RSG + GW (I.P)	[134]
Fenofibrate and Rosiglitazone	Decreasing lung inflammation and down regulating TGF-b1-mediated collagen deposition	BLM-induced PF in rats (Intratracheal instillation of BLM)	No medications Intratracheal instillation of PBS Intratracheal instillation of BLM RSG orally Fenofibrate orally RSG and fenofibrate orally	RSG orally after intratracheal instillation of BLM Fenofibrate orally after intratracheal instillation of BLM RSG and fenofibrate orally after intratracheal instillation of BLM	[135]
Pravastatin	Inhibiting TGF-β1, CTGF, RhoA and cyclin D1 pathways	BLM-induced PF in mice (Intratracheal instillation of BLM)	Intratracheal instillation of Saline Intratracheal instillation of BLM	Intratracheal instillation of BLM plus 30 mg ⁄ kg of pravastatin (I.P.) Intratracheal instillation of BLM plus 300 mg ⁄ kg of pravastatin (I.P.)	[136]
Atorvastatin	Reducing the differentiation of lung fibroblast into myofibroblasts and inducing myofibroblast apoptosis	BLM-induced PF in mice (Intratracheal instillation of BLM)	Treatment of sterile vehicle solutions Intratracheal instillation of BLM (in PBS) ATR in DMSO/PBS (I.P.) for 10d from Day 7	ATR(I.P.) for 10d after 7d from given BLM intratracheally	[137]
Atorvastatin and Ezetimibe	Protecting against hypercholesterolemia-induced lung oxidative stress, inflammation, and fibrosis	Hypercholesterolemia-induced PF in rats	Standard diet(S) Standard diet + 1% cholesterol (SC)	SC with 30 mg/kg/day ATR (I.G.) SC with 10 mg/kg/day ezetimibe (I.G.)	[138]
Probucol	Ameliorating EMT and lung fibrosis through restoration of SIRT3 expression	BLM-induced PF in mice (Intratracheal instillation of BLM)	Intratracheal instillation of BLM and CMC-Na (I.G.) Intratracheal instillation of probucol and probucol (I.G.)	Intratracheal instillation of BLM and probucol plus probucol (I.G.)	[139]

*BLM* Bleomycin, *Sesn2* (Sestrin2)-A stress-inducible protein, *AMPK* AMP-activated protein kinase, *Nrf2* Nuclear factor erythroid 2-related factor 2, *PF* Pulmonary fibrosis, *CMC* Carboxymethyl cellulose, *EMPA* Empaglifozin, *I.P.* Intraperitoneal injection, *I.G.* Oral gavage, *S.C.* Subcutaneous injection; *VEH* 0.9% NaCl solution plus 0.4% acetic acid, *S100A4* fibroblast-specific protein-1(FSP-1), *STAT3* Signal transducer and activator of transcription 3, *PTEN* Chromosome ten, *PQ* Paraquat; *RSG* Rosiglitazone, *GW9662(GW)* a PPAR-γ antagonist, *MET* Metformin, *TGF* Transforming growth factor, *CTGF* Connective tissue growth factor, *ATR* Atorvastatin; Sterile vehicle solutions-Dimethyl sulfoxide/phosphate‐buffered saline (DMSO/PBS) mixture, *EMT* Epithelial-mesenchymal transition, *SIRT3* Sirtuin 3

Moreover, based on preclinical and clinical research data, three major lipid-targeting drugs have been tested in patients with IPF (Table 2. Ref. [140–146]). First, mTOR inhibitors or PI3K/mTOR inhibitors, such as sirolimus (rapamycin) and omipalisib (GSK2126458), have completed randomized, double-blind phase I clinical trials for patients with IPF. Another lipid target of interest is LPA1, which has been shown to mediate fibroblast recruitment [147]. In a phase II clinical trial, the first-generation LPA1 receptor antagonist BMS986020 significantly slowed the decline rate of FVC in patients with IPF, but this trial was prematurely terminated due to an increased risk of hepatic enzyme abnormalities. The second-generation LPA1 receptor antagonist BMS986278 has demonstrated good properties in various preclinical animal experiments [148] and is currently in a phase II clinical trial. The third potential lipid target is ATX. Phase III clinical trials of the ATX antagonist GLPG1690 (ISBELA 1 and 2) to treat IPF were terminated because the benefit-risk profile no longer supported continuing the study. However, other ATX antagonists are still under investigation, such as the drugs BBT-877 and cudetaxestat (BLD-0409), which are poised to enter phase II clinical trials to evaluate their efficacy and safety in patients with IPF.

**Table 2:** Clinical trials of targeted lipid-mediated pathways for pulmonary fibrosis

NCT	Target Specificity	Interventions	Phase	Primary outcome Measures	Enrollment	Allocation	Ref
01462006	mTOR inhibitor	Drug: Sirolimus Other: Placebo	1	Change in peripheral blood concentration of the CXCR4 + fibrocytes up to 22 weeks Number of subjects with drug side-effects up to 22 weeks	32	Randomized Crossover Assignment Quadruple Primary Purpose: Treatment	[140]
01725139	PI3K/mTOR inhibitor	Drug: Omipalisib Other: Placebo	1	PD endpoints pAKT/AKT AUC in blood for GSK2126458 Cmax in blood for GSK2126458 Pre-dose concentration at the end of the dosing interval in blood for GSK2126458 Concentration of GSK2126458 in BALF	17	Randomized Parallel Assignment Double Primary Purpose: Treatment	[141]
03502902	PI3K/mTOR inhibitor	Drug: HEC68498 Other: Placebo	1	Adverse event up to 4 weeks: to assess the safety and tolerability of single dose administered	55	Randomized Parallel Assignment Quadruple Primary Purpose: Treatment	-
01766817	LPA1 receptor antagonist	Drug: BMS-986020 Other: Placebo matching with BMS-986020	2	Change from baseline in FVC rate to week 26	325	Randomized Parallel Assignment Triple Primary Purpose: Treatment	[142]
04308681	LPA1 receptor antagonist	Drug: BMS-986278 Other: BMS-986278 placebo	2	Rate of change in ppFVC in IPF participants up to week 26	278	Randomized Parallel Assignment Triple Primary Purpose: Treatment	[143]
04069143	LPA1 ligand for PET	Diagnostic test: 18F-BMS-986327	1	Incidence of AEs up to 3d after participation Incidence of SAEs up to 30d after participation Radiation dosimetry calculated from PET-CT images 30d after participation Test–retest repeatability 30d after participation Biodistribution and lung uptake calculated from the PET-CT images 30d after participation	14	Non-Randomized Parallel Assignment Open Label Primary Purpose: Diagnostic	-
03711162	ATX inhibitor	Drug: GLPG1690 (ISABELA1) Other: Placebo	3	Annual rate of decline in FVC up to week 52	525	Randomized Parallel Assignment Quadruple Primary Purpose: Treatment	[144]
03733444	ATX inhibitor	Drug: GLPG1690 (ISABELA2) Other: Placebo	3	Annual rate of decline in FVC up to week 52	781	Randomized Parallel Assignment Quadruple Primary Purpose: Treatment	[144]
05483907	ATX inhibitor	Drug: BBT-877 Other: Placebo	2	Reduction in FVC (ml) decline compared to the placebo after 24 weeks of treatment	120	Randomized Parallel Assignment Triple Primary Purpose: Treatment	-
05373914	ATX inhibitor	Drug: BLD-0409 Other: Matching placebo	2	Changes in FVC (L) from Baseline to week 26	200	Randomized Parallel Assignment Quadruple Primary Purpose: Treatment	-
02538536	GPR40 agonist/GPR84 antagonist	Drug: PBI-4050	2	Number of subjects with abnormal laboratory values and/or adverse events that are related to treatment (time Frame: 4 months)	41	N/A Single group Assignment Open Label Primary Purpose: Treatment	[145]
03725852	GPR84 antagonist	Drug: GLPG1205 Other: Placebo	2	Change from baseline in FVC at week 26	68	Randomized Parallel Assignment Quadruple Primary Purpose: Treatment	[146]

NCT Number from https://clinicaltrials.gov/; *PI3K* Phosphoinositide 3-kinase, *Mtor* Mammalian target of rapamycin, *LPA1* Lysophosphatidic acid receptor type1, *ATX* Autotaxin, *GPR* G-protein-coupled receptor, *PD* Pharmacodynamic, *AKT* Protein kinase B,*pAKT* Phosphorylated Akt, *AUC* Area under the curve, *Cmax* Maximum observed concentration, *BALF* bronchoalveolar lavage fluid, *FVC* Forced vital capacity, *ppFVC* Percent predicted forced vital capacity, *AEs* Adverse events, *SAEs* Serious adverse events, *PET-CT* Positron emission tomography-computed tomography

In addition, other lipid-targeting drugs are currently being tested in clinical trials for IPF, such as PBI4050 (a GPR40 agonist and GPR84 antagonist). This drug has completed an open-label phase II clinical trial in IPF patients, demonstrating its safety when used alone or in combination with nintedanib or pirfenidone. Furthermore, GPLG1250 (a functional antagonist of GPR84) has shown antifibrotic effects in animal models and has completed phase II clinical trials. In addition to the targets that have entered testing, many lipid metabolism-related genes that are under investigation, such as Thy-1 (a glycophosphatidylinositol anchored cell surface glycoprotein), SphK1, and S1PL (S1P lyase), have shown promising antifibrotic effects in in vitro or animal experiments and may become new therapeutic targets [149, 150]. In conclusion, further research on the mechanisms of glycemic regulation, lipid modulation and lipid-targeting drugs in pulmonary fibrosis may provide new treatment options for patients with IPF.

### LMRGs are associated with poor prognosis of IPF

To further discuss the relationship between lipid metabolism and IPF prognosis at the gene level, this review provides prognostic analysis results according to LMRGs. These results suggest that high expression levels of multiple LMRGs, which promote lipid accumulation, were associated with a poor survival prognosis in IPF patients. An additional file shows this in more detail (see Additional file 1).

The EFD-related alterations in fat metabolism and secretion explain the negative correlation between excessive VAT and IPF progression, quality of life, and prognosis. This review highlights the benefits of interventions such as NLRP3 inflammasome-targeted therapy to improve ectopic fat tissue dysfunction, anti-aging treatments, aerobic exercise, respiratory muscle strength training, dietary modifications, and even bariatric surgery for IPF patients. Additionally, this review summarized that fat deposition is a common risk factor for both IPF and its pulmonary and extrapulmonary comorbidities. The reported findings suggest that in the majority of IPF patients who currently have limited drug treatment options and are unable to tolerate pulmonary rehabilitation, improving ectopic and visceral fat deposition and managing IPF comorbidities play a key role in optimizing survival quality and extending survival time for IPF patients.

## Strengths and limitations

The major strength of this review is that it provides a new perspective on the pathogenesis and prognosis of IPF. Improving ectopic and visceral fat can contribute to the prevention and treatment of this fatal disease. Furthermore, understanding the molecular mechanisms and signaling pathways of excessive fat deposition-related pulmonary fibrosis is crucial for researchers and drug developers to identify new therapeutic targets. Moreover, the biomarkers, clinical assessment tools, treatments, complications, and prognosis of IPF discussed in this review can improve clinical management. However, there are some limitations in this review. First, differences in study designs and participants make it challenging to extract data for meta-analysis or to give recommendations and guidance based on reliable evidence. Second, it is necessary to continuously track the outcomes of ongoing clinical trials to determine the safety and efficacy of these drugs (hypoglycemic or lipid-lowering drugs, lipid-targeting drugs) in the treatment of IPF. Last, due to a lack of relevant studies, this review cannot provide quantitative thresholds and changes in blood glucose, lipid levels, and fat deposition during the occurrence and development of pulmonary fibrosis.

## Conclusions

In summary, the impact of ectopic and visceral fat deposition on IPF is complex and involves multiple factors, including mechanical injury, lipotoxicity, inflammatory mediators, and insulin resistance. Additionally, ectopic and visceral fat deposition plays a role in various stages of IPF, from onset and exacerbation to complications and prognosis. Current research indicates that medications aimed at improving sugar and lipid metabolism may slow the rate of decline in lung function and reduce the extent of pathological lung fibrosis. Potential therapeutic targets associated with abnormal adipose tissue function have been identified; these targets include the NLRP3 inflammasome, SIRT, and important lipid-related genes linked to IPF.

This review holds great relevance for clinical practice, as it highlights a noticeable correlation between fat deposition and pulmonary fibrosis based on clinical observations. While the six-minute walk test is a commonly employed method in clinical practice to evaluate cardiopulmonary function and prognosis in IPF, it may not be feasible for patients in advanced stages or experiencing acute exacerbations. The review introduces various indicators and tools of body composition analysis that have demonstrated a robust association with lung function and prognosis in pulmonary fibrosis. These noninvasive, easily quantifiable assessment methods offer potential alternatives for evaluating IPF conditions. They pave the way for identifying the necessity for improvements in body fat distribution and exercise capacity, especially in high-risk pulmonary fibrosis patients. Furthermore, this review emphasizes the importance of focusing on the mechanisms of excessive fat deposition in IPF and the latest clinical evidence, which holds promising prospects for the future. This suggests that physicians can potentially prevent and treat IPF by intervening in obesity (through lifestyle interventions and lipid-targeting drugs), addressing sarcopenia (through exercise and pulmonary rehabilitation), and targeting inflammation and LMRGs (via inflammasome modulation and potential gene therapies). However, to gain a deeper understanding of the role of excessive fat deposition in IPF, it is necessary to provide simultaneous assessments of ectopic fat deposition, metabolic status, and the degree of lung fibrosis. Experimental validation of key mechanisms is also essential in future studies. Ultimately, these efforts may lead to the development of novel management or treatment strategies for IPF, the formulation of personalized nutritional and rehabilitation plans, and the significant assessment of lung transplantation risks.

### Supplementary Information

**Additional file 1:** **Supplementary Fig. 1.** The flowchart of literature research selection. **Supplementary Fig. 2.** Bioinformatics evidence of the association between lipid accumulation and poor IPF prognosis.

## Data Availability

Not applicable.

## Abbreviations

IPF: Idiopathic pulmonary fibrosis
EFD: Ectopic fat deposition
NOD: Nucleotide oligomerization domain
NLRP3: NOD-like receptor thermal protein domain associated protein 3
AECs: Alveolar epithelial cells
ROS: Reactive oxygen species
ECM: Extracellular matrix
FDA: Food and Drug Administration
VAT: Visceral adipose tissue
SAT: Subcutaneous adipose tissue
BMI: Body mass index
FAP: Fibro-adipogenic progenitor
ATMs: Adipose tissue macrophages
FFAs: Free fatty acids
COPD: Chronic obstructive pulmonary disease
IR: Insulin resistance
LIF: Lipofibroblast
MYF: Myofibroblast
OSAS: Obstructive sleep apnea syndrome
PH: Pulmonary hypertension
SPA: Surfactant protein A
AT2: Alveolar epithelial type II cells
DLCO: Diffusing capacity of the lungs for carbon monoxide
TLR-2: Toll-like receptor 2
JNK: C-Jun N-terminal kinase
NF-κB: Nuclear factor-kappaB
BLM: Bleomycin
BALF: Bronchoalveolar lavage fluid
TGF-β1: Transforming growth factor beta1
S1P: Sphingosine-1-phosphate
SPHK1: Sphingosine kinase 1
α-SMA: α-Smooth muscle actin
YAP: Yes-associated protein
PAI-1: Plasminogen activator inhibitor-1
EMT: Epithelial-mesenchymal transition
MAPKs: Mitogen-activated protein kinases
Th17: T helper cell 17
IL: Interleukin
AMPK: AMP-activated protein kinase
AGT: Angiotensinogen
RAS: Renin-angiotensin system
Ang II: Angiotensin II
MYF: Myofibroblast
TNF-α: Tumor necrosis factor-alpha
MCP-1: Monocyte chemotactic protein-1
IRS-1: Insulin receptor substrate-1
SIRT: Sirtuin
IFN-γ: Interferon-γ
STAT3: Signal transducer and activator of transcription 3 (STAT3)
CI: Confidence interval
HR: Hazard Ratio
FVC: Forced vital capacity
NLR: Neutrophil-to-lymphocyte ratio
MuRF1: Muscle-specific E3 ubiquitin ligase, muscle RING-finger protein-1
HOMA-IR: Homeostatic Model Assessment of Insulin Resistance
PI3K/mTOR: Phosphoinositide 3-kinase/ mammalian target of rapamycin
FAPs: Fibro-Adipogenic Progenitors
LPA1: Lysophosphatidic acid receptor type1
ATX: Autotaxin
LMRGs: Multiple lipid metabolism-related genes
