# Supplementary material for: Ectopic and visceral fat deposition in aging, obesity, and idiopathic pulmonary fibrosis: an interconnected role
Source: Lipids Health Dis. 2023 Nov 24;22:201. doi: 10.1186/s12944-023-01964-3 (PMC10668383; doi:10.1186/s12944-023-01964-3)
Supplement: Supplementary file 1 — Additional file 1: Supplementary Fig. 1. The flowchart of literature research selection. Supplementary Fig. 2. Bioinformatics evidence of the association between lipid accumulation and poor IPF prognosis. [file 12944_2023_1964_MOESM1_ESM.docx]

**Method**

Search Strategy and Selection Criteria

As of the submission date (September 25, 2023), we conducted a comprehensive search for eligible clinical trials and animal studies using PubMed, ClinicalTrials.gov, Embase, and Cochrane. The primary search terms were "(Lipid-lowering drug) OR (Lipid targeting drug) OR (PCSK9 inhibitor) OR (ezetimibe) OR (statin) OR (DDP4 inhibitor) OR (SGLT2 inhibitor) OR (sulfonylurea) OR (GLP-1 receptor agonists) OR (biguanide)) AND ((pulmonary fibrosis) OR (lung fibrosis))," both separately and in combination. We also manually screened the reference lists of reviews and articles published to identify citations that met the inclusion criteria. The inclusion criteria for the study comprised: (1) Clinical cohorts involving IPF patients or animal experiments of BLM-induced pulmonary fibrosis models or obesity-associated pulmonary fibrosis models; (2) Interventions primarily focusing on hypoglycemic, lipid-lowering, or lipid-targeting drugs; (3) Reporting any of the following outcomes: exercise capacity measured by 6MWD, pulmonary function, lung CT, lung fibrosis pathology or Masson's staining, and markers of lung fibrosis (hydroxyproline, collagen, α-SMA, TGF-β, etc.). The exclusion criteria included: (1) Reviews, conference abstracts; (2) Fibrosis of other organs; (3) Different forms of the same drug formulation, e.g., nanoparticles; (4) Only reporting side-effects of drugs; (5) Cell experiment or high-throughput drug screening; (6) Combined with other adjunctive therapies (e.g., simvastatin combined with vitamin D3); (7) Subjects with muscle wasting or obesity but without lung fibrosis.

Results of selection

A total of 1,469 articles were initially retrieved from our search. After removing 84 duplicates and excluding 1,341 articles based on title and abstract, 38 articles remained. After full-text review, 21 papers met the inclusion criteria. Supplementary Figure 1 summarizes the selection process.


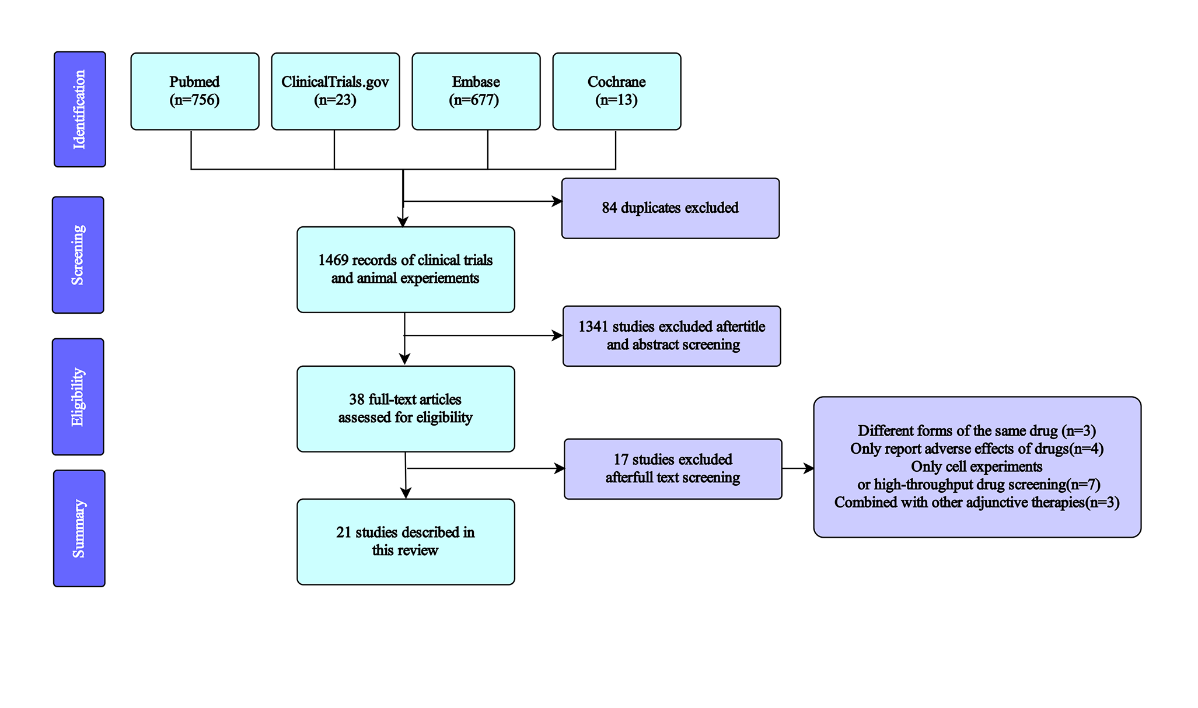


Supplementary Fig.1 The flowchart of literature research selection

### 8. Multiple lipid metabolism-related genes (LMRGs) are associated with poor prognosis of IPF

We obtained the GSE70866 gene expression dataset from the GEO database (https://www.ncbi.nlm.nih.gov/geo/), which comprised 176 IPF samples and 20 normal control samples. From this dataset, we identified differentially expressed genes (DEGs) (Fig. 3 A) and extracted upregulated DEGs with a log fold change (logFC) > 1 and an adjusted p-value < 0.05. The list of LMRGs was obtained from a published work [131]. A Venn diagram (Fig.3 B) displayed the 16 intersecting genes between the DEGs and LMRGs. We represented the expression of these 16 intersecting genes in IPF and non-IPF samples using a heatmap (Fig.3 C). To gain further insights into the biological functions of these 16 genes, we performed biological process Gene Ontology (GO) enrichment analysis using the Metascape database (https://metascape.org), with the criteria set as follows: Min Overlap = 3, P Val Cutoff = 0.01, and Min Enrichment = 1.5. The results revealed a strong association with pathways related to fatty acid metabolism, lipid biosynthesis, lipid homeostasis, and hypoxia, among others (Supplementary Fig.2). Additionally, Kaplan-Meier survival curves.


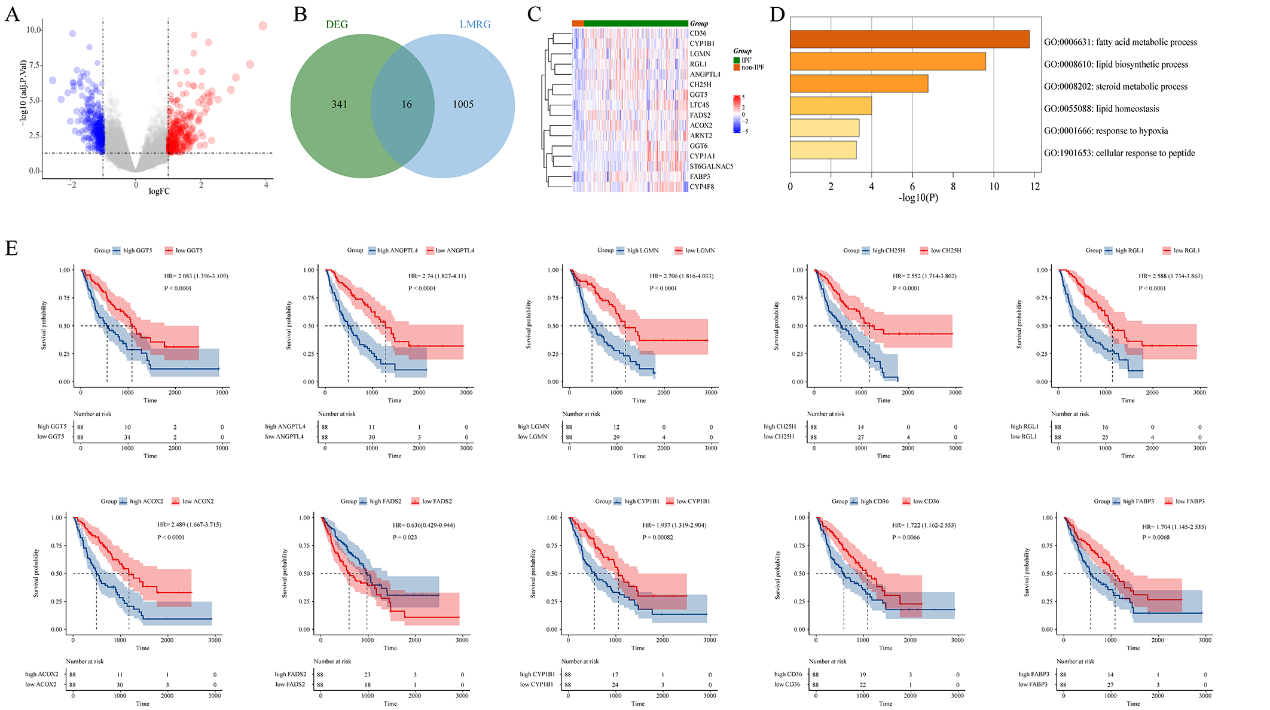


Supplementary Fig.2 Bioinformatics evidence of the association between lipid accumulation and poor IPF prognosis. (A) the volcano plot of DEGs between IPF and non-IPF; (B) the Venn diagram identified 16 intersecting genes; (C) the heatmap showed the expression difference of intersecting genes in IPF and non-IPF; (D) Biological process GO enrichment analysis of intersecting genes; (E) Kaplan-Meier analysis of LMRGs in IPF.
